# Anti-inflammatory effects of ethanolic extract from *Abeliophyllum distichum* (*Miseon* Tree) leaves in mice with dextran sulfate sodium-induced ulcerative colitis

**DOI:** 10.29219/fnr.v69.11052

**Published:** 2025-07-21

**Authors:** Hye-Jung Moon, Youn-Soo Cha, Kyung-Ah Kim

**Affiliations:** 1 Department of Food Science and Human Nutrition, Jeonbuk National University, Jeonju, Republic of Korea; 2 K-Food Research Center, Jeonju, Republic of Korea; 3 Department of Food and Nutrition, Chungnam National University, Daejeon, Republic of Korea

**Keywords:** Abeliophyllum distichum, dextran sulfate sodium, colitis, NF-kappa B

## Abstract

*Abeliophyllum distichum* (Miseon tree), a native Korean plant, is known for the high phenolic content in its leaves. The ethanolic leaf extracts of *A. distichum* have shown antioxidant, anti-obesity, and anti-inflammatory properties. However, studies on its potential to improve colitis are limited. This study aimed to determine whether the ethanolic extract of *A. distichum* leaves (ADE) could alleviate dextran sulfate sodium (DSS)-induced colitis in mice. The mice were divided into three groups, and the experimental group was given 300 mg/kg ADE for 4 weeks. One week before the end of the experiment, 3% DSS was added to the drinking water to induce colitis. The clinical symptoms of colitis and damage to colon tissue, including the increase in *Enterobacteriaceae* abundance and a decrease in *Bifidobacterium* in the colon, were evaluated during DSS treatment. DSS overactivated the nuclear factor (NF)-κB signaling pathway, resulting in excessive production of pro-inflammatory cytokines. In contrast, ADE alleviated the DSS-induced colitis symptoms, protected against colonic tissue damage, and restored the balance of *Enterobacteriaceae* and *Bifidobacterium* levels in the colon. Moreover, ADE effectively inhibited the DSS-induced overactivation of the NF-κB signaling pathway within the colon and mitigated abnormal inflammatory responses. These findings suggest that ADE protects against colitis by modulating the growth of some intestinal strains and the NF-κB pathway in the colon, supporting its potential as a natural agent.

## Popular scientific summary

- Extract from *Abeliophyllum distichum* leaves improved DSS-induced colon tissue damage and clinical symptoms of colitis.
- Extract of *Abeliophyllum distichum* leaves was observed to alter the population of specific gut commensal bacteria and suppress the overactivated NF-κB signaling pathway in the colon, which resulted in the recovery of excessive cytokine levels in the serum and colon, thereby alleviating colitis.

A chronic inflammation of the mucosal layer extending from the rectum to the colon is known as ulcerative colitis (UC) (1). Clinical characteristics of UC include diarrhea, abdominal pain, bloody diarrhea, rectal urgency, and weight loss. UC affects various organs, including the skin, joints, biliary tract, and eyes, causing extraintestinal symptoms (2). The occurrence of UC began with the industrialization and westernization of society. In the 20th century, the prevalence of UC was high in Western nations, including North America, Europe, and Oceania. However, in the 21st century, it has spread to Asia, Africa, and South America, posing a threat to global health (3). However, the exact mechanism of UC pathogenesis remains unclear, and it can result from alterations in the balance of intestinal bacteria, damage to the mucosal barrier, and disruptions in immune regulation by environmental variables and genetic susceptibility (4, 5).

Current therapeutic agents for UC include aminosalicylates, corticosteroids, immunomodulators, biologics, and small-molecule drugs to suppress excessive inflammatory responses. However, these drugs are associated with adverse reactions and high treatment costs (6–8). In addition, UC carries a cumulative relapse risk of 70–80% after 10 years, and despite drug treatment, the remission rate is only 20–30%, making it challenging to treat (9–11). These factors have led to an ongoing effort to develop newer, more effective treatments for UC that are less prone to showing adverse reactions (6, 8). For example, based on Ingenuity Pathway Analysis conducted on patients with UC, interleukin (IL)-6, IL-1β, and tumor necrosis factor (TNF)-α, and nuclear factor (NF)-κB were identified as potential key regulators of inflammation (12). In particular, the targeting of the NF-κB signaling pathway is a key approach for treating UC, and numerous studies have evaluated natural products that target this pathway (6, 13, 14).

*Abeliophyllum distichum* (known in Korea as *Miseon* tree) is a deciduous shrub native to the Korean Peninsula and is protected by the Korean government (15). However, with recent advancements in the mass propagation of the *A. distichum*, various studies have been conducted on its fruits, flowers, stems, and leaves (16–19). The leaves of *A. distichum* contain various phenolic compounds, such as chlorogenic acid, caffeic acid, rutin, ferulic acid, and verbascoside (also known as acteoside) as well as polyphenolic compounds such as gallic acid, taxifolin, narirutin, and rutin (18, 20). The 70% ethanol extract of *A. distichum* leaves (ADE) demonstrated stronger inhibition of androgen receptor signaling than did the extracts from distilled water and 95% hexane, indicating its potential to prevent prostatic hyperplasia (21). ADE has also been shown to ameliorate obesity by lowering the expression of genes linked to adipogenesis in adipose tissue *in vivo* (22). Moreover, ADE have anti-inflammatory effects via suppressing lipopolysaccharide (LPS)-induced TNF-α production by inhibiting mitogen-activated protein kinase (MAPK)/NF-κB signaling in mouse macrophages (20). However, despite its widely researched anti-inflammatory properties, few studies have investigated the ability of *A. distichum* to ameliorate UC.

Therefore, in the present study, we investigated the potential of ADE to alleviate inflammation in mice with UC induced by dextran sulfate sodium (DSS). Specifically, we focused on the effects of ADE on the NF-κB signaling pathway, a critical regulator of inflammation, to evaluate its potential as a natural agent for alleviating colitis.

## Materials and methods

### Sample preparation

The ADE samples used in this experiment were provided by Korea Prime Pharmacy Co., Ltd. (Gwangju, Korea) and were prepared as described in a previous study (23). In summary, ADE samples were prepared by extraction at 75°C for 4 h with a 20-fold addition of 70% ethanol to dried ADE, followed by filtration, concentration, and lyophilization.

### Animals

Male C57BL/6J mice, 5-week-old, were obtained from Central Lab Animal, Inc. (Seoul, Republic of Korea). Animals were acclimated for 6 days under consistent conditions, including a temperature of 22 ± 2°C, humidity of 50 ± 5%, and 12-h light/12-h dark cycles. After acclimation, 21 mice were divided into three experimental groups (*n* = 7 per group): Nor group with untreated normal control mice; DSS group with DSS-treated control mice; and ADE group containing mice treated with DSS and ADE. All animal experiments were approved by the Animal Ethics Committee of Chungnam National University (IACUC approval number: 202112-CNU-214).

### Sample treatment and colitis induction in mice

Samples were administered by oral gavage to mice for 29 days. In the ADE group, ADE was orally administered at 300 mg/kg body weight daily. Phosphate-buffered saline (PBS) was supplied in the same amounts to the Nor and DSS groups. On day 21, UC was induced by replacing the drinking water of the DSS and ADE groups with a 3% DSS solution (w/v; MW: 36,000–50,000 Da, MP Biochemical, Solon, OH, USA) and allowing them to drink for 6 days.

After the day following the conclusion of the DSS drinking period, the fecal samples were collected. All mice were euthanized, and blood and colon tissue were obtained. Colon tissues were measured in length and weight, some were fixed in 4% formalin, and the remaining colon tissues were stored at −80°C.

### Evaluation of the severity of colitis

The severity of UC was assessed using the disease activity index (DAI), a system that scores body weight loss, stool consistency, and gross bleeding over the DSS administration. The DAI score ranged from 0 to 9, with weight loss scores of 0 (< 1%) to 3 (> 10%), stool formation scores of 0 (none) to 3 (watery diarrhea), and blood in stool scores of 0 (none) to 3 (gross bleeding).

### Histological studies

Hematoxylin and eosin (H&E) staining was used for histopathological analysis of colon tissue. Colon tissue sections embedded in paraffin after fixation in 4% formalin were stained with H&E and observed under a light microscope (DM2500; Leica Microsystems, Wetzlar, Germany) installed in the Center for University Research Facilities (CURF) at Jeonbuk National University (Jeonju, Korea).

### Fecal microbial population

The fecal samples were diluted in sterile PBS and plated onto the respective selective media: *Bifidobacterium*, blood liver agar (BL agar, MBcell, Seoul, Republic of Korea), at 37°C for 48 h; *Enterobacteriaceae*, desoxycholate agar (MBcell), at 37°C for 24 h. Results were expressed as Colony Forming Units per gram of feces.

### Measurement of pro-inflammatory cytokines

Enzyme-linked immunosorbent assay (ELISA) kits were employed to analyze pro-inflammatory cytokines in both colon tissue and serum. The manufacturer’s guidelines were followed in order to quantify the levels of TNF-α (Invitrogen, Vienna, Austria), IL-1β, and IL-6 (R&D Systems, Minneapolis, MN, USA).

### Messenger RNA (mRNA) analysis by Quantitative real-time polymerase chain reaction (PCR) (qRT-PCR)

TRIZol reagent (Takara Bio, Inc.) was utilized to extract total RNA from the colon. The extracted total RNA was then synthesized into cDNA using PrimeScript RT Master Mix (Takara Bio, Inc.). The gene expression was quantified employing a 7500 Real-Time PCR System (Applied Biosystems, Foster City, CA, USA). The relative expression of the target gene was normalized to glyceraldehyde 3-phosphate dehydrogenase (GAPDH) expression, a housekeeping gene.

### Western blotting

The colon tissue was homogenized and centrifuged to obtain a supernatant. The supernatant was used for western blotting analysis to confirm the expression of the target protein. Primary antibodies were used at a dilution of 1:1,000 for NF-κB phosphorylated-p65 (p-p65), NF-κB p65, induced nitric oxide synthase (iNOS), cyclooxygenase-2 (COX-2), and β-actin (Cell Signaling Technology, Danvers, MA, USA). Secondary antibodies of horseradish peroxidase-labeled anti-rabbit or anti-mouse were used at a 1:3,000 dilution (Cell Signaling Technology). The protein bands on the prepared membranes were visualized using a ChemiDoc system (ATTO LuminoGraph II, ATO, Tokyo, Japan). Signals were quantified using ImageJ software (National Institute of Health, Bethesda, Maryland, USA).

### Statistical analysis

All results were expressed as mean ± standard deviation (SD). Statistical analysis was performed utilizing SPSS 18.0 software (SPSS Inc., Chicago, IL, USA). Differences among groups were evaluated for significance using a one-way analysis of variance (ANOVA) followed by Duncan’s post hoc tests at a significance level of *P* < 0.05.

## Results

### Alleviatory effects of ADE on the clinical symptoms of colitis

**Fig. 1:**
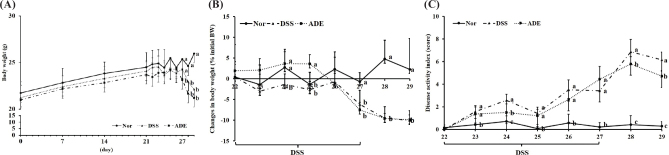
Effect of the 70% ethanol extract of *Abeliophyllum distichum* leaves (ADE) on colitis symptoms. (A) Body weight changes during the entire experimental period. (B) Body weight changes during DSS administration. (C) DAI during DSS administration. Data are presented as mean ± standard deviation (*n* = 7). The letters next to the values indicate significant differences at the *P* < 0.05 level (a > b > c; *P* < 0.05). Nor, normal group; DSS, dextran sulfate sodium group; ADE, a group treated with ADE at 300 mg/kg body weight along with DSS administration.

The DSS and ADE groups exhibited significant reductions in body weight in comparison with that of the Nor group by the end of the experiment (Fig. 1A, B). Additionally, the DAIs in both the DSS and ADE groups were higher than those in the Nor group from 1 day after DSS administration until the end of the DSS administration period (Fig. 1C). However, after cessation of DSS administration, the ADE group demonstrated faster recovery from colitis symptoms than did the DSS group, as indicated by a reduction in the DAI for 2 days.

### Effects of ADE on the length-to-weight ratio of the colon and the histopathological changes in colitis

**Fig. 2:**
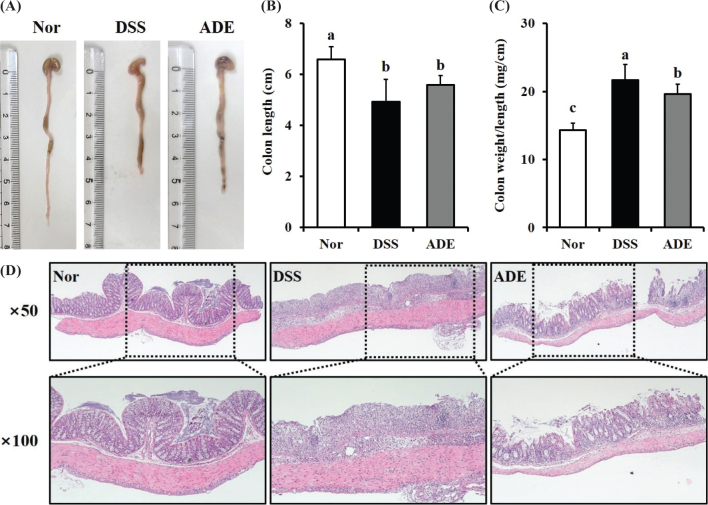
Effects of the 70% ethanol extract of *Abeliophyllum distichum* leaves (ADE) on DSS-induced colon tissue damage. (A) Representative images of colonic length for each group. (B) Statistical analysis of colonic length. (C) Statistical analysis of colon weight-to-length ratio. (D) Representative hematoxylin and eosin images. Data are presented as mean ± standard deviation (*n* = 7). Lowercase letters above the bars indicate significant differences at the *P* < 0.05 level (a > b > c). Nor, normal group; DSS, dextran sulfate sodium group; ADE, a group treated with ADE at 300 mg/kg body weight along with DSS administration.

The effect of ADE on colon length was confirmed when colitis was induced (Fig. 2A–C). Colon length in the DSS group was significantly shortened to approximately 25% of that in the Nor group. While the colon length in the ADE group showed no significant difference from that in the DSS group, it showed a tendency to increase by approximately 13% (Fig. 2A, B). The colon weight-to-length ratio in the DSS group was 21.66 mg/cm, a significant increase of 51.15% from that of the Nor group (14.33 mg/cm). However, the corresponding value in the ADE group was 19.61 mg/cm, which was 9.46% lower than that in the DSS group (Fig. 2C).

The effect of ADE on the histopathology of the colon tissue after induction of colitis was also observed (Fig. 2D). The DSS group exhibited the histopathological features of colitis, including crypt loss, destruction of goblet cells, and infiltration of inflammatory cells into the mucosal and submucosal layers. Furthermore, thickening of the submucosa and muscle layer was observed, along with epithelial cell destruction. However, the ADE group demonstrated relief from DSS-induced colon tissue damage, including inflammatory cell infiltration into the mucosa and submucosa, as well as crypt loss.

### Effects of ADE on the commensal bacterial population in colitis

**Fig. 3:**
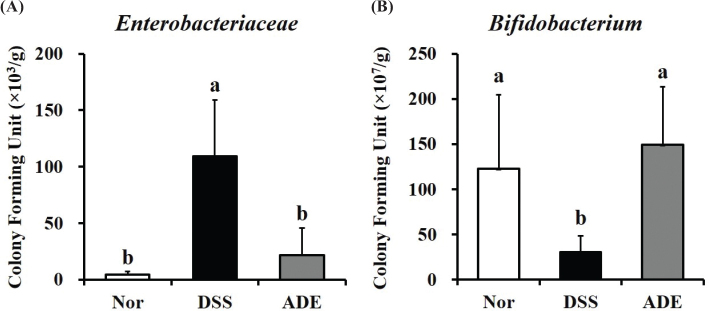
Effects of the 70% ethanol extract of *Abeliophyllum distichum* leaves (ADE) on the populations of *Enterobacteriaceae* and *Bifidobacterium* in the feces of mice with DSS-induced colitis. (A) *Enterobacteriaceae* and (B) *Bifidobacterium* counts in feces. Data are presented as mean ± standard deviation (*n* = 6). Lowercase letters above the bars indicate significant differences at the *P* < 0.05 level (a > b > c). Nor, normal group; DSS, Dextran sulfate sodium group; ADE, a group treated with ADE at 300 mg/kg body weight along with DSS administration.

Changes in the commensal bacteria in the colon have been reported to cause colitis (24, 25). Changes in *Enterobacteriaceae* and *Bifidobacterium* were confirmed in the feces when ADE was administered during colitis induction with DSS (Fig. 3A, B). In comparison with that of the Nor group, the DSS group showed a significant increment of approximately 24-fold in the abundance of *Enterobacteriaceae* and a 4-fold reduction in the abundance of *Bifidobacterium*. However, the ADE group showed a reduction in the abundance of *Enterobacteriaceae* and an increase in the abundance of *Bifidobacterium* to levels similar to those in the Nor group.

### Effects of ADE on the pro-inflammatory cytokines in colitis

Alterations in pro-inflammatory cytokines were observed in the colon and serum of ADE-treated mice (Table 1). The levels of TNF-α and IL-6 in the colon tissue, as well as TNF-α, IL-6, and IL-1β in the serum, were significantly higher in the DSS group than in the Nor group. Conversely, the ADE group demonstrated a significant reduction in the levels of all pro-inflammatory cytokines that were elevated in the DSS group, thereby inhibiting the progression of inflammation.

**Table 1:** Effects of the 70% ethanol extract of *Abeliophyllum distichum* leaves (ADE) on pro-inflammatory cytokines in the colon and serum in DSS-induced colitis mice

Parameters		Nor	DSS	ADE
Serum (pg/mL)	TNF-α	1.82 ± 0.53^c^	7.89 ± 1.25^a^	5.54 ± 1.80^b^
	IL-1β	0.05 ± 0.03^b^	0.08 ± 0.04^a^	0.03 ± 0.01^b^
	IL-6	0.45 ± 0.05^b^	2.65 ± 1.20^a^	1.15 ± 0.33^b^
Colon (pg/μg protein)	TNF-α	28.76 ± 25.71^b^	64.88 ± 22.50^a^	32.68 ± 15.34^b^
	IL-1β	2.85 ± 0.71^c^	37.32 ± 14.19^a^	25.28 ± 7.52^b^
	IL-6	6.92 ± 0.57^a^	7.18 ± 1.06^a^	6.57 ± 1.37^a^

Data are presented as mean ± standard deviation (*n* = 7). The letters next to the values indicate significant differences at the *P* < 0.05 level (a > b > c; *P* < 0.05). ADE, a group treated with ADE at 300 mg/kg body weight along with DSS administration.

### Effects of ADE on mRNA and protein expression related to the NF-κB signaling pathway in colitis

**Fig. 4:**
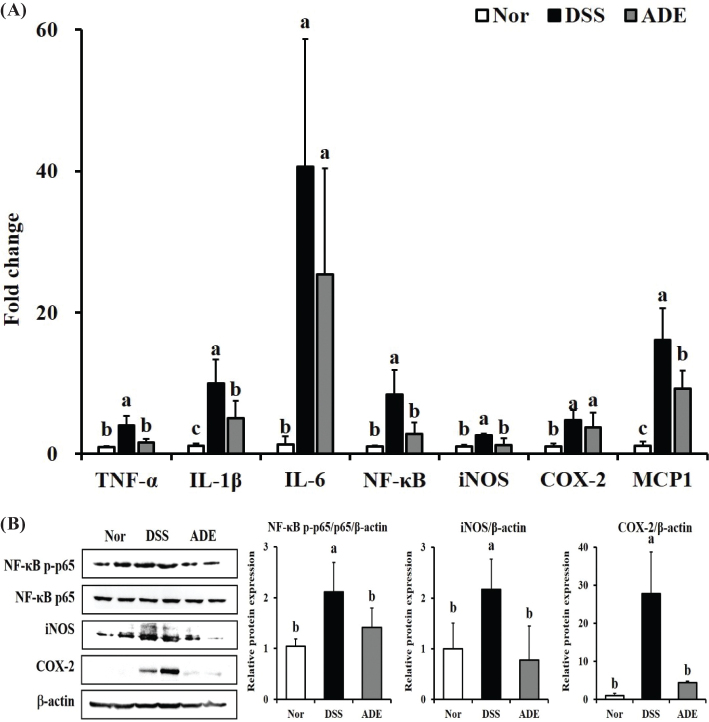
Effects of the 70% ethanol extract of *Abeliophyllum distichum* leaves (ADE) on the expression of genes and proteins related to the NF-κB signaling pathway in the colon of DSS-induced colitis mice. (A) Gene expression and (B) protein expression related to the NF-κB signaling pathway in colon tissue. Data are presented as mean ± standard deviation (*n* = 7). Lowercase letters above the bars indicate significant differences at the *P* < 0.05 level (a > b > c). Nor, normal group; DSS, dextran sulfate sodium group; ADE, a group treated with ADE at 300 mg/kg body weight along with DSS administration.

The NF-κB signaling pathway has been suggested to be a major signaling pathway related to colitis (26). The effects of ADE on mRNA and protein expression related to the NF-κB signaling pathway were examined (Fig. 4A). The DSS group showed significantly upregulated mRNA expression of the pro-inflammatory cytokines TNF-α, IL-1β, and IL-6 in comparison with the Nor group. Additionally, the assessment of inflammation-related factors showed significantly elevated mRNA expression of NF-κB, iNOS, COX-2, and monocyte chemoattractant protein (MCP)-1 in the DSS group. In contrast, the ADE group showed significantly lower inflammation-related mRNA expression levels than the DSS group.

Moreover, in the DSS group, NF-κB p-p65 was excessively activated, and the expression levels of the downstream inflammation-related enzymes iNOS and COX-2 were also increased (Fig. 4B, C). Conversely, the ADE group showed inhibition of the activity of NF-κB p-p65 that was increased by DSS, thereby reducing the expression of iNOS and COX-2. Therefore, ADE alleviates excessive inflammatory reactions by inhibiting the overactive NF-κB signaling pathway during colitis.

## Discussion

UC treatment aims to achieve immediate symptom relief and promote mucosal healing, ultimately enhancing the patient’s overall quality of life (27). We investigated whether ADE, a natural product endemic to the Korean Peninsula that has shown anti-inflammatory effects on macrophages (20), has the potential to prevent colitis in mice. Although ADE did not inhibit the body weight loss in DSS-induced colitis mice, it significantly reduced the DAI. ADE has been previously shown to prevent obesity (22), suggesting that it may not influence body weight recovery. However, the anti-inflammatory effects of ADE are thought to ameliorate symptoms such as rectal bleeding and diarrhea. Thus, the anti-inflammatory effects of ADE may have reduced damage to colon tissue, thereby ensuring recovery from symptoms such as rectal bleeding and diarrhea.

Additionally, UC is characterized by pathological features such as goblet cell depletion and neutrophil inflow, and morphological changes such as shortened colon length (28) and ADE improved these symptoms. Verbascoside and chlorogenic acid, major components of ADE, have also been shown to improve colon length, mucosal damage, and infiltration of inflammatory cells in mice with colitis (18, 23, 29, 30). Thus, the polyphenols in the ADE may serve as the potential components for preventing colitis.

Inflammatory bowel diseases (IBDs) such as UC are characterized by an increase in *Enterobacteriaceae* and a decrease in *Bifidobacterium* (31, 32). We found that ADE contributed to the restoration of this balance. This result is similar to those obtained with dietary resveratrol administration in DSS-induced colitis mice and could be attributed to the increased abundance of beneficial bacteria, such as *Bifidobacterium* and *Lactobacillus* that protect against tissue damage from potentially harmful bacteria, such as *Enterobacteriaceae* (33). However, further research is required to understand the impact of ADE on the overall gut microbiota and the improvement of colitis.

A healthy intestine maintains intestinal immune homeostasis through appropriate interactions between the resident microbial community and the host immune system. In contrast, UC occurs when the balance of the colonic mucosal immune system is disrupted due to overactivation of the transcription factor NF-κB, which is caused by various factors, such as bacterial LPSs, pro-inflammatory cytokines, and viruses (26, 34). NF-κB consists of five subunits, of which p65 can directly activate the transcription of the target gene (26, 34). Activated NF-κB promotes the gene transcription of pro-inflammatory cytokines (e.g. IL-1β, IL-6, and TNF-α), as well as inflammation-related factors (e.g. iNOS and COX-2) (14). In addition, excessive production of cytokines and chemokines (e.g. MCP-1) resulting from NF-κB activation mediates the infiltration of inflammatory cells such as macrophages and neutrophils into damaged colon tissues, exacerbating colitis (35–37).

In this study, ADE inhibited the activation of NF-κB p-p65, which was typically heightened in the DSS group. This inhibition affected the expression of downstream genes and proteins, consequently contributing to the alleviation of colitis by reducing the concentration of inflammatory cytokines in both the colon and serum. Curcumin has been considered as a promising phytonutrient for treating IBD, with no observed side effects in animal studies and clinical trials (38). Similarly, ADE has demonstrated potential as a natural agent to prevent colitis. Based on these findings, further studies and subsequent clinical trials to identify the optimal concentration and bioactive compounds responsible for the colitis-protective effect of ADE may contribute to its development as a natural therapeutic agent.

## Conclusions

ADE reversed the altered gut microbiota composition observed in colitis by reducing the abundance of *Enterobacteriaceae* while increasing that of *Bifidobacterium*. Moreover, ADE exhibits anti-inflammatory properties by mitigating pro-inflammatory cytokines through the suppression of the highly activated NF-κB signaling pathway, which is elevated during UC. Consequently, ADE has the potential as natural materials for the management of colitis.
